# Supplementary material for: MetaRibo-Seq measures translation in microbiomes
Source: Nat Commun. 2020 Jun 29;11:3268. doi: 10.1038/s41467-020-17081-z (PMC7324362; doi:10.1038/s41467-020-17081-z)
Supplement: Supplementary file 10 — Supplementary Data 7 [file 41467_2020_17081_MOESM10_ESM.zip › File2/Confidence_High_Taxonomy/415567_out.krona.html]

Javascript must be enabled to view this page.

members
magnitude
magnitudeUnassigned
count
unassigned
taxon
rank

415567\_out

63

superkingdom
63
2

phylum
1
1798710

1906119
order
1


SRS063190\_contig\_number\_9584
1916221
1
species

61
phylum
976

200643
class
61

171549
order
61

family
2
171550

239759
genus
2


SRS019910\_contig\_number\_7344SRS1041039\_contig\_number\_1557
28117
2
species

2005525
family
1

genus
1
375288


SRS044535\_contig\_number\_11081
46503
species
1

58
family
815

816
genus
58

33
species

SRS011302\_contig\_number\_15685SRS012849\_contig\_number\_26634SRS013382\_contig\_number\_contig-100\_501.23189SRS014415\_contig\_number\_913SRS014534\_contig\_number\_834SRS015663\_contig\_number\_27744SRS015782\_contig\_number\_26240SRS017433\_contig\_number\_8823SRS019178\_contig\_number\_3124SRS019285\_contig\_number\_contig-100\_111.112SRS023583\_contig\_number\_3406SRS023715\_contig\_number\_20366SRS043841\_contig\_number\_2938SRS047044\_contig\_number\_1142SRS049712\_contig\_number\_22653SRS050026\_contig\_number\_2934SRS051031\_contig\_number\_16031SRS054352\_contig\_number\_18192SRS055017\_contig\_number\_31263SRS064232\_contig\_number\_608SRS065397\_contig\_number\_9860SRS078242\_contig\_number\_10764SRS078665\_contig\_number\_2329SRS1041091\_contig\_number\_17167SRS1041138\_contig\_number\_10379SRS1041157\_contig\_number\_1280SRS105082\_contig\_number\_13728SRS143070\_contig\_number\_7700SRS143181\_contig\_number\_3488SRS143876\_contig\_number\_23228SRS147653\_contig\_number\_2182SRS148319\_contig\_number\_3987SRS893285\_contig\_number\_1153
310297


SRS147271\_contig\_number\_24992
1263052
1
species

species
1
46506

SRS077589\_contig\_number\_665

23
species

SRS014534\_contig\_number\_47SRS015217\_contig\_number\_8061SRS018817\_contig\_number\_18241SRS018817\_contig\_number\_contig-100\_338.146859SRS019381\_contig\_number\_11903SRS020508\_contig\_number\_37SRS044535\_contig\_number\_10970SRS049995\_contig\_number\_12147SRS064973\_contig\_number\_118SRS065397\_contig\_number\_1381SRS076876\_contig\_number\_248SRS078176\_contig\_number\_12476SRS098881\_contig\_number\_6519SRS105153\_contig\_number\_7375SRS1055034\_contig\_number\_8410SRS1055056\_contig\_number\_9483SRS145308\_contig\_number\_877SRS146764\_contig\_number\_15000SRS146813\_contig\_number\_1254SRS147425\_contig\_number\_701SRS893327\_contig\_number\_7901SRS893341\_contig\_number\_9146SRS893369\_contig\_number\_8251
820

1239
phylum
1

1
class
186801

1
order
186802

family
1
541000

1
genus
1263


SRS011239\_contig\_number\_18690
1637508
1
species
